# The 150 most important questions in cancer research and clinical oncology series: questions 40–49

**DOI:** 10.1186/s40880-017-0222-7

**Published:** 2017-07-13

**Authors:** Chinese Journal of Cancer

**Affiliations:** Editorial Office of Chinese Journal of Cancer, Sun Yat-sen University Cancer Center, Guangzhou, 510060 Guangdong P. R. China

**Keywords:** Mouse models, Cervical cancer, Anti-metastasis drug, Cachexia, Oligo-metastasis, Warburg effect, Epstein–Barr virus, Lung cancer

## Abstract

Since the beginning of 2017, *Chinese Journal of Cancer* has published a series of important questions in cancer research and clinical oncology, which sparkle diverse thoughts, interesting communications, and potential collaborations among researchers all over the world. In this article, 10 more questions are presented as followed. Question 40. Why do mice being used as tumorigenesis models raised in different places or different conditions possess different tumor formation rate? Question 41. How could we generate more effective anti-metastasis drugs? Question 42. What is the molecular mechanism underlying heterogeneity of cancer cachexia in patients with the same pathologic type? Question 43. Will patients with oligo-metastatic disease be curable by immunotherapy plus stereotactic body radiotherapy? Question 44. Can the Warburg effect regulation be targeted for cancer treatment? Question 45. Why do adenocarcinomas seldom occur in the small intestine? Question 46. Is Epstein–Barr virus infection a causal factor for nasal natural killer/T cell lymphoma formation? Question 47. Why will not all but very few human papillomavirus-infected patients eventually develop cervical cancer? Question 48. Why do cervical carcinomas induced by human papilloma virus have a low mutation rate in tumor suppressor genes? Question 49. Can viral infection trigger lung cancer relapse?

## Text

Since the beginning of 2017, *Chinese Journal of Cancer* has published a series of important questions in cancer research and clinical oncology [1–7], which sparkle diverse thoughts, interesting communications, and potential collaborations among researchers all over the world. In this article, Questions 40–49 are selected and presented. This program of collecting and publishing the key questions is still ongoing. Please send your thoughtful questions to Ms. Ji Ruan via email: ruanji@sysucc.org.cn.

## **Question 40:** Why do mice being used as tumorigenesis models raised in different places or different conditions possess different tumor formation rate?

### Background and implications

Under experimental conditions, researchers often encounter a problem of unstable tumor formation rate using mice models even with the same tumor cell line, especially when the housing conditions are changed. Actually, to overcome the problems caused by individual variations, we usually use a group of animals (e.g., 5–10 mice) to obtain an average measurement for statistical analysis. It has been speculated that the genomic instability of cancer cells might contribute to the variation in tumor formation rate. However, host factors influenced by the housing environment might also contribute to the variation. Determining the interacting mechanisms between housing conditions and tumor formation rate will be very helpful to cancer prevention in human populations.

#### Submitter

Jiangchao Li.

#### Affiliation and email

School of Basic Courses, Guangdong Pharmaceutical University, Guangzhou, Guangdong, P. R. China.

Lijiangchao1234@163.com.

## **Question 41:** How could we generate more effective anti-metastasis drugs?

### Background and implications

Today, most cancer deaths are caused by metastasis, not primary tumor growth. Our understanding on tumor metastasis is accumulating; however there is few anti-metastatic drugs approved for clinical practice. Most existing drugs are approved for their properties of inhibiting cancer cell proliferation. Generating potential drugs that block metastasis is important for the development of oncological therapy, by which patients might gain survival benefit.

#### Submitter

Dong-Fang Meng.

#### Affiliation and email

Sun Yat-sen University Cancer Center, Guangzhou, Guangdong, P. R. China.

mengdf@sysucc.org.cn.

## **Question 42:** What is the molecular mechanism underlying heterogeneity of cancer cachexia in patients with the same pathologic type?

### Background and implications

Cancer cachexia is a lethal alteration in some cancer patients. Multiple neuroendocrine factors are believed to be involved in modulating the metabolic rates responsible for cancer cachexia. Some hormones released by cancer cells or neuroendocrine glands are known to manipulate the metabolic rates. However, patients with cancer of the same pathologic type usually show heterogeneous manifestations of cancer cachexia with undetermined mechanism(s). Unveiling these underlying mechanism(s) will be very helpful for better surveillance on the risk of cancer cachexia and effective therapy for correcting cancer cachexia and prolonging patient survival.

#### Submitter

Zixun Zeng.

#### Affiliation and email

Medical School of Sun Yat-sen University, Guangzhou, Guangdong, P. R. China.

zengzx@mail2.sysu.edu.cn.

## **Question 43:** Will patients with oligo-metastatic disease be curable by immunotherapy plus stereotactic body radiotherapy?

### Background and implications

With the advances in cancer immunotherapy (from interleukin-2 [IL-2] to cytotoxic T lymphocyte-associated antigen-4 [CTLA-4]/programmed death-1 [PD-1]/programmed death ligand 1 [PD-L1] inhibitors), excellent durable responses are observed in patients with metastatic disease treated with immunotherapy. A good example is that patients with metastatic melanoma treated with immunotherapy comprising of CTLA-4 and/or PD-1 inhibitors have shown promising durable responses. Recent evidence has also demonstrated that adding local radiotherapy, especially stereotactic body radiotherapy (SBRT), can enhance the effects of immunotherapy and then more abscopal effects are observed. Oligo-metastasis is a state of which cancer patients have limited metastatic tumor burden. Understanding the molecular mechanism(s) and interaction(s) of specific immunotherapy and SBRT, especially the mechanisms underlying abscopal therapeutic effects upon the interaction of these two treatment modalities, may be very helpful in providing possible control (make cancer a chronic disease) or even cure in patients with oligo-metastatic disease from various types of malignancy.

#### Submitter

Bin S. Teh.

#### Affiliation and email

Houston Methodist Hospital, Cancer Center and Research Institute, Houston, Texas, USA.

bteh@houstonmethodist.org.

## **Question 44:** Can the Warburg effect regulation be targeted for cancer treatment?

### Background and implications

Most cancer cells predominantly produce energy by a high rate of glycolysis followed by lactic acid fermentation in the cytosol, rather than by a comparatively low rate of glycolysis followed by oxidation of pyruvate in mitochondria as in most normal cells. This Warburg effect provides energy and metabolic intermediates needed for production of proteins, lipid, and nucleotide acids in cancer cells. Successful inhibition of the functions of critical regulators of the Warburg effect may largely decrease current cancer mortality.

#### Submitter

Zhimin Lu.

#### Affiliation and email

MD Anderson Cancer Center, Houston, Texas, USA.

zhiminlu@mdanderson.org.

## **Question 45:** Why do adenocarcinomas seldom occur in the small intestine?

### Background and implications

The incidence of gastrointestinal adenocarcinomas is very high in the whole world. However, most of these adenocarcinomas occur in the stomach and large intestine. As to the small intestine, it seems having some bodyguards to prevent epithelial cells from cancer initiation, resulting in a very low incidence of adenocarcinoma in the small intestine in human being. Even the ampullary cancer, which arises from the ampulla vater and manifests in the duodenum, is usually originated from the pancreas or bile duct. In human being, loss-of-function mutations in the adenomatous polyposis coli (*APC*) gene have been found to be an oncogenic factor for colorectal cancer. Interestingly, in the mouse with mutants in the *Apc* gene, named *Min* (multiple intestinal neoplasia) mouse, adenoma and adenocarcinoma can be found in both the small intestine and colon. Clearly, there should be some “secret weapons” for the human epithelium of the small intestine to avoid carcinogenesis. To reveal the underlying structural and molecular mechanism(s) may be very helpful for preventing gastric and colorectal adenocarcinomas, and even adenocarcinomas in the lung, female reproductive system, and other organs.

#### Submitter

Qingping Jiang.

#### Affiliation and email

Department of Pathology, Third Affiliated Hospital, Guangzhou Medical University, Guangzhou, Guangdong, P. R. China.

Jiangqp2003@126.com.

## **Question 46:** Is Epstein–Barr virus (EBV) infection a causal factor for nasal natural killer/T-cell lymphoma (NKTCL) formation?

### Background and implications

Extranodal nasal NKTCL is a rare and aggressive malignancy that occurs predominantly in Asian and Latin American populations. EBV infection is a known risk factor. Moreover, EBV latent membrane protein 1 (LMP1), the best documented oncoprotein of the EBV latent gene products, has been shown to be expressed in most NKTCLs and be associated with poor patient prognosis. However, convincing evidence has not been presented yet to show the causal effect of EBV infection on the tumorigenesis of NKTCL. Clarification of the causal role of EBV infection in the tumorigenesis of NKTCL by using animal model will be very helpful for preventing NKTCL and developing new therapeutics for this fatal disease.

#### Submitters

Qingqing Cai, Yu (Sharon) Fang.

#### Affiliation and email

Department of Medical Oncology, Sun Yat-sen University Cancer Center, Guangzhou, Guangdong, P. R. China.

caiqq@sysucc.org.cn.

## **Question 47:** Why will not all but very few human papillomavirus (HPV)-infected patients eventually develop cervical cancer?

### Background and implications

HPV infection is considered the main etiologic factor of cervical cancer. While most viral infections are cleared spontaneously by the host immunity, very few persist and eventually cause cancer. The progression from persistent HPV infections to the development of cancer generally takes decades, providing excellent time window for precision prevention and treatment of the disease. To seize these opportunities, a deep understanding of the interplay between HPV and its host cancer cells must be gained. Identifying the key molecular markers that are involved in HPV-related cervical carcinogenesis will help to construct a prediction model, which could be used for precise recognition of HPV-infected patients who are going to regress/progress. This knowledge may be further translated into clinical precision medicine to reduce the huge economic burden posed by cervical screening programs and HPV vaccination programs.

#### Submitter

Hu Zheng.

#### Affiliation and email

The First Affiliated Hospital of Sun Yat-sen University, Guangzhou, Guangdong, P. R. China.

huzheng5@mail.sysu.edu.cn.

## **Question 48:** Why do cervical carcinomas induced by human papilloma virus have a low mutation rate in tumor suppressor genes?

### Background and implications

It is clear that HPV is a key carcinogen in cervical carcinomas, and HPV DNA exists in more than 95% of tumors. Recent studies have even revealed that the HPV genome can integrate into the accessible regions of the human genome, in which over 1500 integration sites have been detected, and consequently induce significant alteration in the gene expression profile responsible for carcinogenesis. However, the mutations of other well-studied tumor suppressors including P53 are not common in cervical cancers. As one of the hallmarks of cancer cell, intrinsic genomic instability in HPV-induced cervical cancer is not as remarkable as that in other cancer types. Revealing the underlying molecular mechanism(s) may be very helpful to explain an alternative approach to carcinogenesis independent to conventional intrinsic genomic instability in cervical cancer. So does it in other HPV-related cancers.

#### Submitters

Jieping Chen, Jihong Liu.

#### Affiliation and email

Department of Gynecologic Oncology, Sun Yat-sen University Cancer Center, Guangzhou, Guangdong, P. R. China.

chenjp@sysucc.org.cn.

## **Question 49:** Can viral infection trigger lung cancer relapse?

### Background and implications

Cold and seasonal flu caused by viruses are the most common respiratory disease. Importantly, many lung cancer patients have a history of frequent colds before lung cancer relapse. It is unclear whether viral infections could trigger lung cancer relapse. A speculation of the involvement of immune function in lung cancer relapse has been proposed based on the observations that some tumor-associated immune factors, e.g., interleukin (IL)-2, IL-6, IL-8, associate with poor patient prognosis. Particularly, IL-8 has been confirmed to be able to promote metastasis in a variety of human cancers, including lung cancer. Theoretically, the expression of these tumor-associated immune factors can also be up-regulated by viral infection. Upon further validation by epidemiologic studies to confirm the close relationship between viral infection and lung cancer relapse, explorations for better understanding of the determinants that activate cancer dormancy by viral infection may provide useful knowledge to prevent lung cancer relapse.

#### Submitter

Lan Huang.

#### Affiliation and email

The First Affiliated Hospital of Zhengzhou University, Zhengzhou, Henan, P. R. China.

huanglan@hotmail.com.
